# Future Impact of Various Interventions on the Burden of COPD in Canada: A Dynamic Population Model

**DOI:** 10.1371/journal.pone.0046746

**Published:** 2012-10-11

**Authors:** Mehdi Najafzadeh, Carlo A. Marra, Larry D. Lynd, Mohsen Sadatsafavi, J. Mark FitzGerald, Bruce McManus, Don Sin

**Affiliations:** 1 Department of Medicine, Harvard Medical School, Boston, Massachusetts, United States of America; 2 Faculty of Pharmaceutical Sciences, University of British Columbia, Vancouver, British Columbia, Canada; 3 Centre for Health Evaluation and Outcome Sciences, St. Paul's Hospital, Vancouver, British Columbia, Canada; 4 Division of Respiratory Medicine, University of British Columbia, Vancouver, British Columbia, Canada; 5 Department of Pathology and Laboratory Medicine, University of British Columbia, and The Prevention of Organ Failure Center of Excellence, Vancouver, British Columbia, Canada; 6 The Institute for Heart and Lung Health (the UBC James Hogg Research Laboratories), Vancouver, British Columbia, Canada; Clinica Universidad de Navarra, Spain

## Abstract

**Background:**

Chronic obstructive pulmonary disease (COPD) is a growing economic burden worldwide. Smoking cessation is thought to be the single most effective way of reducing the economic burden of COPD. The impact of other strategies such as interventions that predict risk of disease, reduce progression of disease, or reduce exacerbations has not been systematically studied.

**Objectives:**

We estimated the economic and clinical burden of COPD over the next 25 years in Canada and the impact of three potential interventions (screening test for predisposition to COPD, new drugs to avoid progression into more severe disease stages, and predictive test for exacerbations) on COPD burden.

**Methods:**

Using a dynamic simulation model, we projected the total burden of COPD (cost, morbidity, and mortality) from 2011 to 2035 using the population of Canada as a case study. The model stratified population based on sex, age, smoking status, respiratory symptoms, and their COPD stage. The cost and quality adjusted life years (QALYs) associated with each intervention were estimated.

**Results:**

The model indicates that annual societal cost of COPD is $4.52 billion (B) Canadian dollars in 2011 and will reach $3.61B ($7.33B undiscounted) per year in 2035. Over the next 25 years, COPD will be responsible for approximately $101.4B in societal costs ($147.5B undiscounted) and 12.9 million QALYs lost (19.0 million undiscounted). Our results suggested that the best strategy to reduce the financial burden of COPD is by reducing exacerbations. Smoking cessation, while it is the cornerstone of COPD prevention, has only a modest effect in attenuating the financial burden of COPD over the next 25 years in Western countries such as Canada.

**Conclusion:**

Our data suggest that any intervention that can reduce the number of exacerbations has a substantial impact on morbidity and costs of COPD and should be considered in conjunction with the ongoing efforts to reduce smoking rates.

## Introduction

The human and economic burden of chronic obstructive pulmonary disease (COPD) is substantial and is rapidly increasing worldwide [1]. COPD is now the fourth leading cause of mortality globally [2] and the third leading cause in the US [3]. Given the enormous burden of COPD across the world, there is significant interest in developing population-based programs to address this global crisis. It is widely believed that smoking cessation is the single most effective intervention for COPD [4]. However, the impact of other strategies such as those that control progression, or reduce exacerbations has not been systematically evaluated. In particular, new discoveries about molecular biomarkers that can provide information about predisposition, diagnosis, and prognosis of COPD have raised the possibility of utilizing these potential biomarkers to reduce burden of COPD [5]–[7]. Although clinical usefulness of these biomarkers remains to be validated, better understanding about their potential impact on COPD is valuable. For instance, by comparing the long term clinical and economic outcomes of using these molecular tests, we can rank strategies based on their potential benefit at the population level.

In the current study, we evaluated the impact of three potential interventions that targeted different stages of the disease using hypothetical molecular tests. For this purpose, we first developed a dynamic model that projected the total burden of COPD (epidemiology, cost, morbidity, and mortality) over the next 25 years using the population of Canada as a case study. Then, by measuring the clinical and economic outcomes, we estimated the incremental cost and incremental effectiveness of using each potential intervention and compared the results across the three interventions. Finally, we discuss the implications of our findings for prioritization of interventions to reduce the global burden of COPD.

## Methods

### 1. Structure of the model

**Figure 1:**
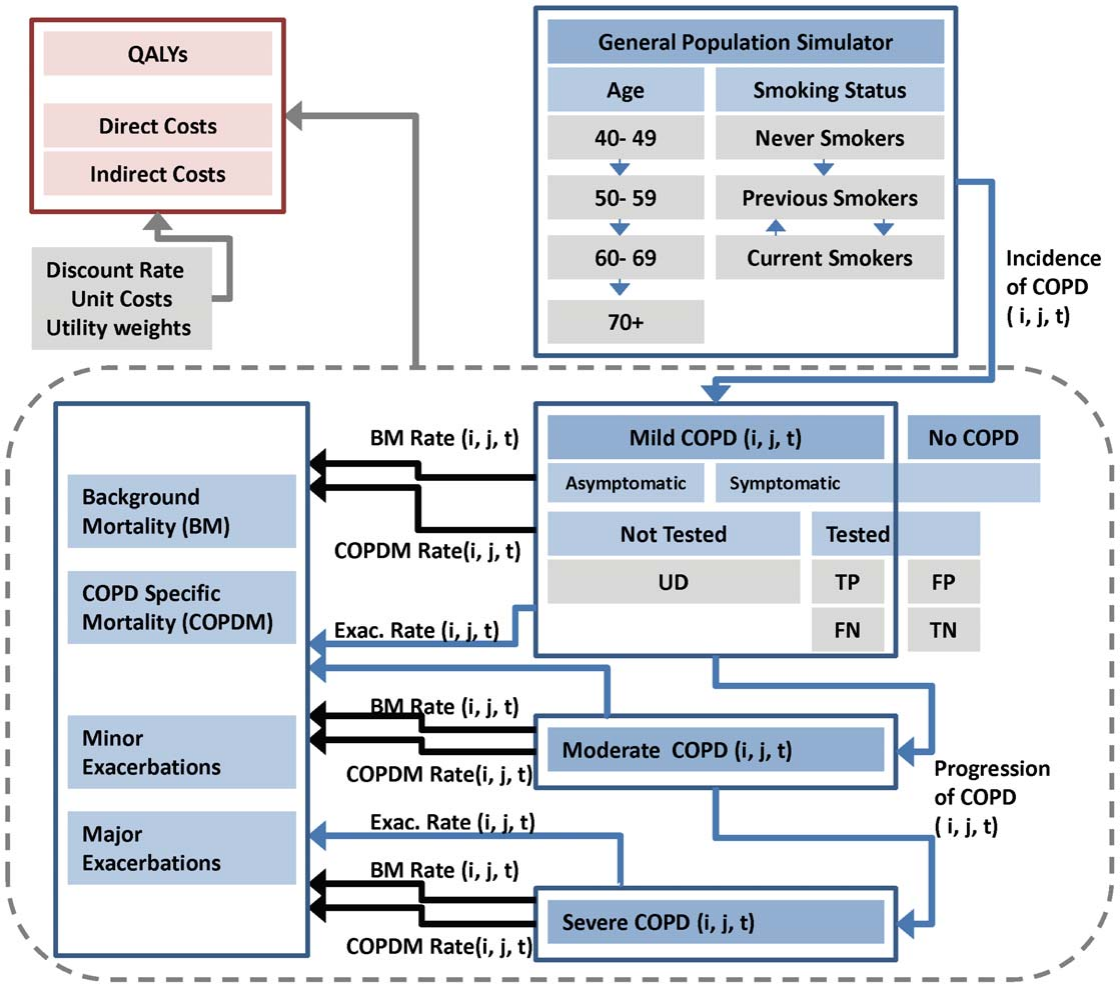
Model structure. Footnotes: i: 40–49, 50–59, 60–69, 70+. j: Current Smoker, Previous Smoker, Never Smoker. t: Time. UD: Undiagnosed; TP: True Positive; TN: True Negative; FP: False Positive; FN: False Negative. The model ran separately for men and women.

Based on data from the existing literature and population growth projections from Statistics Canada [8], the present study built a system-wide dynamic simulation model using Vensim® PLE Plus Version 5.10e (Harvard, MA, USA) to predict the future burden of COPD (Figure 1). The dynamic model used demographics data, which took into account projected annual rates of birth, immigration, emigration, and mortality from 2011 to 2035, of Canadians 40 years or older (40–49, 50–59, 60–69, ≥70 years)[8]. The Canadian population for each age group was further divided into based on sex (men, women), smoking status (current smokers, previous smokers, and non smokers), presence of respiratory symptoms (symptomatic vs. asymptomatic), and COPD severity stage (no COPD, mild, moderate, or severe).

The prevalence estimates for each disease severity of COPD were based on data from the BOLD study [9], which ascertained estimates of COPD in the general population of individuals 40 years and older using post-bronchodilator spirometry. Since many subjects in the BOLD who demonstrated significant airflow limitation (i.e. COPD) had never been diagnosed clinically with COPD, these estimates included both diagnosed and undiagnosed COPD. We followed the Global initiative for chronic Obstructive Lung Disease (GOLD) classification scheme (in accordance with BOLD study) to divide COPD patients into three levels of disease severity: mild (GOLD I), moderate (GOLD II), and severe (GOLD III or IV).

Spirometry is the currently accepted method for diagnosing COPD and determining disease severity (based on patients' forced expiratory volume in one second (FEV1) values). However, in practice, spirometry is not always used to make the diagnosis. In these cases, diagnosis is based on clinical judgement. Thus, we assumed that only a percentage of symptomatic individuals would undergo spirometry testing. Given the actual prevalence of COPD in each sub-group (based on BOLD study) and the performance characteristics of spirometry (i.e., sensitivity and specificity), the model estimated sub-groups with a true positive (TP), false negative (FN), false positive (FP), and true negative (TN) diagnosis of COPD based on spirometry results. The sub-group with undiagnosed COPD (UD) was also included in the model. This sub-group (i.e. UD) consisted of mild asymptomatic COPD patients and mild symptomatic COPD patients who did not undergo spirometry.

All TP, FN, and UD patients were initially classified as mild COPD; however, UD and FN cases were assumed to be correctly diagnosed (TP) if they progressed into the moderate or severe stages of disease (i.e., there were no FN or UD patients with moderate or severe COPD as symptoms would lead to a diagnosis of COPD). Progression rates across the different stages of the disease (mild, moderate, and severe) were calculated based on changes in lung function related to aging and smoking status [10] (Table 1). Individuals were able to move between different sub-groups in the model (e.g. moving from younger age group to the next age group, or moving from current smoker to previous smoker subgroups) depending on the natural pathways and potential interventions defined in the model. The effect of smoking cessation was modeled as transition from the current sub-group into previous smoker sub-group. Therefore, subsequent disease trajectories were modified according to parameters that corresponded to previous smokers.

**Table 1:** Key input parameters in the model.

	No COPD	Mild	Moderate	Severe	Reference
Background annual mortality rates (per 10,000)					Estimated^a^, Appendix S2
Men					
Current smokers					
40–49	27.0	27.0	27.0	27.0	
50–59	69.0	69.0	69.0	69.0	
60–69	186.0	186.0	186.0	186.0	
70+	830.7	830.7	830.7	830.7	
previous smokers					
40–49	18.9	18.9	18.9	18.9	
50–59	48.3	48.3	48.3	48.3	
60–69	130.2	130.2	130.2	130.2	
70+	581.5	581.5	581.5	581.5	
Never smokers					
40–49	16.2	16.2	16.2	16.2	
50–59	41.4	41.4	41.4	41.4	
60–69	111.6	111.6	111.6	111.6	
70+	498.4	498.4	498.4	498.4	
Women					
Current smokers					
40–49	16.8	16.8	16.8	16.8	
50–59	43.0	43.0	43.0	43.0	
60–69	108.9	108.9	108.9	108.9	
70+	694.8	694.8	694.8	694.8	
Previous smokers					
40–49	11.8	11.8	11.8	11.8	
50–59	30.1	30.1	30.1	30.1	
60–69	76.2	76.2	76.2	76.2	
70+	486.4	486.4	486.4	486.4	
Never smokers					
40–49	10.1	10.1	10.1	10.1	
50–59	25.8	25.8	25.8	25.8	
60–69	65.3	65.3	65.3	65.3	
70+	416.9	416.9	416.9	416.9	
Prevalence of respiratory symptoms (%)					Estimated using Mannino et al [36] b, Appendix S2
Men					
Current Smokers	36.7	95.1	95.1	95.1	
Previous smokers	19	49.2	49.2	49.2	
Never smokers	14.5	37.6	37.6	37.6	
Women					
Current Smokers	40	100	100	100	
Previous smokers	24.7	64.1	64.1	64.1	
Never smokers	21.8	56.5	56.5	56.5	
Spirometry test uptake rate	37%	-	-	-	Camp et al [12]
Sensitivity of spirometry test	92%	-	-	-	Schneider et al [37]
Specificity of spirometry test	84%	-	-	-	Schneider et al [37]
Distribution of COPD stages in Men (%)					Estimated^c^, Appendix S2
Current smokers					
40–49	79.3	17.0	3.4	0.4	
50–59	74.4	17.0	7.7	0.8	
60–69	67.0	17.0	14.5	1.6	
70+	48.0	17.0	31.6	3.4	
Previous smokers					
40–49	81.4	15.2	3.0	0.3	
50–59	77.1	15.2	6.9	0.7	
60–69	70.4	15.2	13.0	1.4	
70+	53.4	15.2	28.3	3.0	
Never smokers					
40–49	89.6	8.5	1.7	0.2	
50–59	87.2	8.5	3.9	0.4	
60–69	83.5	8.5	7.2	0.8	
70+	74.0	8.5	15.8	1.7	
Distribution of COPD stages in Women (%)					Estimated^c^, Appendix S2
Current Smokers					
40–49	72.3	24.4	2.5	0.8	
50–59	72.3	24.4	2.5	0.8	
60–69	47.9	24.4	20.9	6.8	
70+	22.5	24.4	40.0	13.1	
Previous Smokers					
40–49	90.7	8.2	0.8	0.3	
50–59	90.7	8.2	0.8	0.3	
60–69	82.5	8.2	7.0	2.3	
70+	73.9	8.2	13.5	4.4	
Never Smokers					
40–49	91.9	7.1	0.7	0.2	
50–59	91.9	7.1	0.7	0.2	
60–69	84.9	7.1	6.1	2.0	
70+	77.5	7.1	11.6	3.8	
Exacerbation rates (per patient year)	-	0.79	1.22	1.47	Spencer et al [13]
Proportion of minor exacerbations (%)	-	94	93	90	Spencer et al [13]
Proportion of major exacerbations (%)	-	6	7	10	Spencer et al [13]
Probability of death per major exacerbation (%)	-	4.6	4.6	4.6	Estimated using Camp et al [12] d
Average progression time into the next COPD stage (years), Men					Estimated^e^, Appendix S2
Current smokers	-	22	16	-	
Previous smokers	-	30	21	-	
Never smokers	-	30	21	-	
Average progression time into the next COPD stage (years), Women					Estimated^e^, Appendix S2
Current smokers	-	22	17	-	
Previous smokers	-	32	21	-	
Never smokers	-	32	21	-	
Utilities					f
40–49	0.874				Johnson et al [17]
50–59	0.864				
60–69	0.828				
70–79	0.79				
Chronic stage (all ages)		0.81	0.72	0.67	Spencer et al [13]
Minor exacerbation (all ages)		0.72	0.658	0.475	Spencer et al [13]
Major exacerbation (all ages)		0.519	0.447	0.408	Spencer et al [13]
Direct costs(2011 Can$)					g
Maintenance		144	430	628	Spencer et al [13]
Per minor exacerbation episode		161	161	161	Spencer et al [13]
Per major exacerbation episode		6,501	6,501	6,501	Spencer et al [13]
Total direct cost per patient		572	1167	1796	Estimated, Appendix S2
Indirect costs (2011 Can$)					
Maintenance		36	215	524	Estimated using Chapman et al, Spencer et al [13], [20], Appendix S2
Minor exacerbation episode		40	80	134	
Major exacerbation episode		1625	3250	5417	
Total indirect cost per patient		143	583	1497	Estimated^h^, Appendix S2

a rates were calculated based on relative risk of mortality per smoking status [25], 2002 Canadian life tables, and 2010 mortality estimates, Statistics Canada (See Appendix S2).

b were estimated based on the symptom rates among smokers, previous smokers, and never smokers reported by Mannino et al [36] for men and women and also proportion of patients without COPD reported by Buist et al [9].

c Estimated based on the reported rates for men and women in Buist et al [9] (See Appendix S2).

d Estimated based on COPD specific mortality rate of 30.4 per 10,000 (Camp et al [12]) and probabilities of major exacerbations.

e Estimated based on progression rates in Hoogendoorn et al [31] (See Appendix S2).

f these weights are EQ-5D Scores.

g Costs in in Mannino et al [36] were multiplied by 1.155 to reflect the changes in Canadian Consumer Price Index (CPI) between 2002 and 2011.

h Estimated based on proportion of major/minor exacerbations used in Spencer et al [13].

### 2. Model Assumptions

#### A. Input Parameters

There is significant variability in the estimated prevalence of COPD across various studies owing in large part to the use of different case definitions. For this analysis, we used the GOLD criteria for diagnosis [11]. Based on this definition, the prevalence of mild, moderate, and severe COPD is reported to be 11.1% (SE 1.2), 7.3% (SE 1.0), and 0.9% (SE 0.3) in adults 40 years of age or older, respectively in the sample of the population in Vancouver, Canada [9]. We considered these estimates to be representative of rates across Canada [12]. We used sub-group specific prevalence rates in the model in order to account for differences in COPD prevalence related to age, sex, smoking status, and COPD severity stage (Table 1). The sub-group specific incidence rates of mild COPD were then calculated based on the sub-group specific prevalence rates and changes in the size (i.e. number of individuals) in each sub-group. Therefore, the incidence of mild COPD in each sub-group was proportional to the changes in the population in that sub-group (i.e. sub-group specific prevalence rates of mild COPD did not change by time). Incidence rates of moderate and severe COPD in each sub-group were then determined in the model based on the transition rates from mild to moderate and from moderate to severe stages (Table 1).

Exacerbations are major drivers of COPD morbidity and mortality. We modeled the likelihood of exacerbations according to disease severity (Table 2). The annual rates of minor (defined as an increase in symptoms requiring office visits and treatment with oral corticosteroids or antibiotics) and major exacerbations (defined as an increase in symptoms requiring an emergency visit or hospitalization) in each sub-group were projected based on published exacerbation rates and the number of COPD cases at any given time with a specific level of disease severity [13], [14].

**Table 2:** Outcomes of hypothetical interventions.

Intervention	Target population	Intervention effect size (effect on annual rates)	Effect on total # of COPD (million)	Effect on # of exacerbations (million)	Effect on # of COPD deaths (thousand)	Total costs (billion dollar)	Total QALYs lost (million)	Incremental cost (billion)	Incremental QALY lost (million)	Monetary Benefit (billion)
No Intervention	-		5.89	6.12	19.06	97.06	13.37	-	-	-
I	Early smokers	−10%	5.86	6.09	18.99	96.87	13.35	0.18	0.02	1.22
		−25%	5.82	6.05	18.87	96.59	13.31	0.47	0.05	3.17
		−50%	5.71	5.94	18.53	95.65	13.21	1.41	0.16	9.39
II	All COPD patients	−10%	5.89	6.07	18.77	95.65	13.04	1.41	0.32	17.61
		−25%	5.89	6.01	18.33	93.54	12.56	3.52	0.81	43.80
		−50%	5.90	5.89	17.60	90.09	11.77	6.97	1.59	86.69
III	All COPD patients	−10%	5.91	5.53	17.25	90.25	12.61	6.81	0.75	44.46
		−25%	5.95	4.64	14.49	79.96	11.47	17.09	1.89	111.78
		−50%	6.02	3.13	9.79	62.60	9.54	34.46	3.83	225.72

I. Decreasing smoking start rate (by testing for COPD predisposition in early smokers).

II. Decreasing progression rates (by access to new pharmacogenomic agents).

III. Decreasing exacerbations (by prediction of exacerbators).

#### B. Mortality

The background mortality rates were assumed to be related to age and smoking status. The mortality rate from COPD is estimated to be 10,000 deaths per year in Canada (30.4 per 100,000 persons)[12]. In the model, it was assumed that any COPD related death was associated with a major exacerbation. As such, the impact of smoking status, age, and disease severity on COPD-related mortality was captured indirectly by their effect on exacerbations (Table 1).

#### C. Quality of life

The model captured COPD related morbidity by using quality adjusted life years (QALYs). We assumed that COPD had a negative impact on patients' quality of life and that it was proportional to disease severity (Table 1). We also assumed that exacerbations reduced quality of life for the patient. The impact of COPD on patients' quality of life was captured using the EQ-5D utility weights [15], [16]. Age specific EQ-5D utility weights derived from the general Canadian population [17] were used as reference utility weights to measure the area under the curve of QALYs lost due to COPD. Deaths were converted to equivalent QALY losses by assuming that the quality of life of patients dropped to zero at the time of death. The model calculated the overall QALYs lost at the population level, incorporating the number of COPD deaths, the number of COPD cases (mild, moderate, and severe), and the number of minor/major exacerbations over time (Table 1). We assumed that the quality of life of UD and FN patients was affected by COPD (similar to a TP patient).

#### D. Costs

The direct annual cost of COPD and exacerbations were modeled differentially based on COPD disease severity. Using previously derived data [13], we modeled the direct costs of maintenance therapy for the projected number of patients at each stage of the disease. Direct costs of exacerbations were also calculated based on the estimated unit cost per minor/major exacerbations [13] (Table 1) and the frequency of exacerbation episodes throughout the simulation. We assumed that patients with mild COPD who were undiagnosed (UD) or misdiagnosed (FN) did not generate any maintenance costs, while all TP and FP cases were assumed to accrue maintenance costs (e.g. costs related to drug treatment). However, we assumed that all COPD patients, including UD and FN cases, were at risk of experiencing an exacerbation and we incorporated associated costs with exacerbation for those cases [18], [19]. We also considered indirect costs related to COPD, which accounts for approximately 20%, 33%, and 45% of total costs for mild, moderate, and severe COPD patients respectively. [20] (Table 1)

#### E. Model perspective

The time horizon of the simulation was from 2011 to 2035. We populated the model to simulate the burden of COPD in Canada, and as such, Canadian unit costs were used to estimate the economic burden of COPD. However, exchange rates between Canadian and US dollar have also been reported to facilitate some basic comparisons. The costs and QALYs were discounted at 3% annual discount rate in the base case scenario. However, the effects of other discount rates were also captured in the sensitivity analysis. The unit costs reported in earlier studies [13] were adjusted using Consumer Price Index (CPI) in Canada to approximate 2011 unit costs.

### 3. Model validation and sensitivity analysis

The model was initially calibrated to replicate observed population trends as predicted by Statistics Canada [8]. The structure of the model and input parameters was carefully discussed with expert clinicians and the model was validated by comparing the predicted epidemiological variables against the predictions in a number of independent studies. Furthermore, the results were examined under several extreme scenarios to assure robustness and internal validity of the model. Extensive one way sensitivity analyses were conducted to evaluate the effect of variation in input parameters on the results. Since long-standing asthma may be a risk factor for COPD, we also modeled the possible impact of the rising rates of asthma on the costs of COPD over time.

### 4. Modeling of interventions

We estimated the outcomes for the following three hypothetical interventions.

### 5. Hypothetical intervention I: Test for COPD predisposition

Some studies suggest that repeated medical advice of physicians and educating individuals on the risk of COPD or other diseases related to cigarette smoking can significantly increase the probability of smoking cessation [21], [22]. In this context, access to a screening test that predicts one's risk of developing COPD in early smokers may result in a significant benefit [23]. We modeled the effect of a hypothetical molecular screening test that could be utilized to assess risk of developing COPD. We assumed that this preventive intervention would be used to screen those who just have started smoking (incident cases).

### 6. Hypothetical intervention II: New pharmacogenomic interventions to reduce progression into more severe disease stages

Studies for detecting treatment effect directly on clinical outcomes (mainly phase II clinical trials) need long follow-up, which is a major limitation for developing new treatments for COPD [24]. Detecting novel molecular biomarkers that can function as surrogate endpoints for characterizing COPD and its progression could advance and accelerate the drug discovery process [6], [21].

We modeled the effect of a hypothetical pharmacogenomic intervention that could reduce (but not reverse) the rate of disease progression. As such, all patients with mild or moderate COPD would remain in their current stage for a longer period of time under this scenario.

### 7. Hypothetical intervention III: Predictive test for exacerbations

Unscheduled physician visits and hospitalizations for exacerbations are responsible for more than 60% of direct medical costs of COPD [20]. Prediction of exacerbations (or exacerbators) in advance could potentially reduce the risk of exacerbations through the use of targeted treatments. In this scenario, we assumed that a hypothetical molecular test could accurately predict exacerbators and therefore by using appropriate interventions we could reduce the frequency of exacerbations.

## Results

### 1. Base case projections

**Figure 2:**
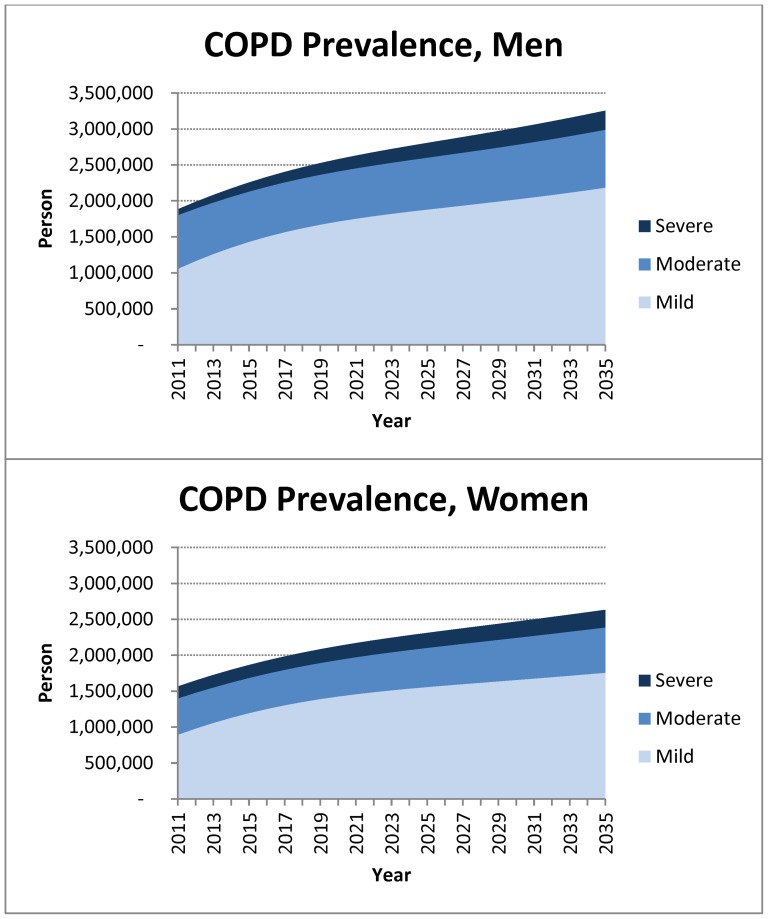
The projected increase in the prevalence of COPD in Canada in men (A) and women (B) across disease severities.

**Figure 3:**
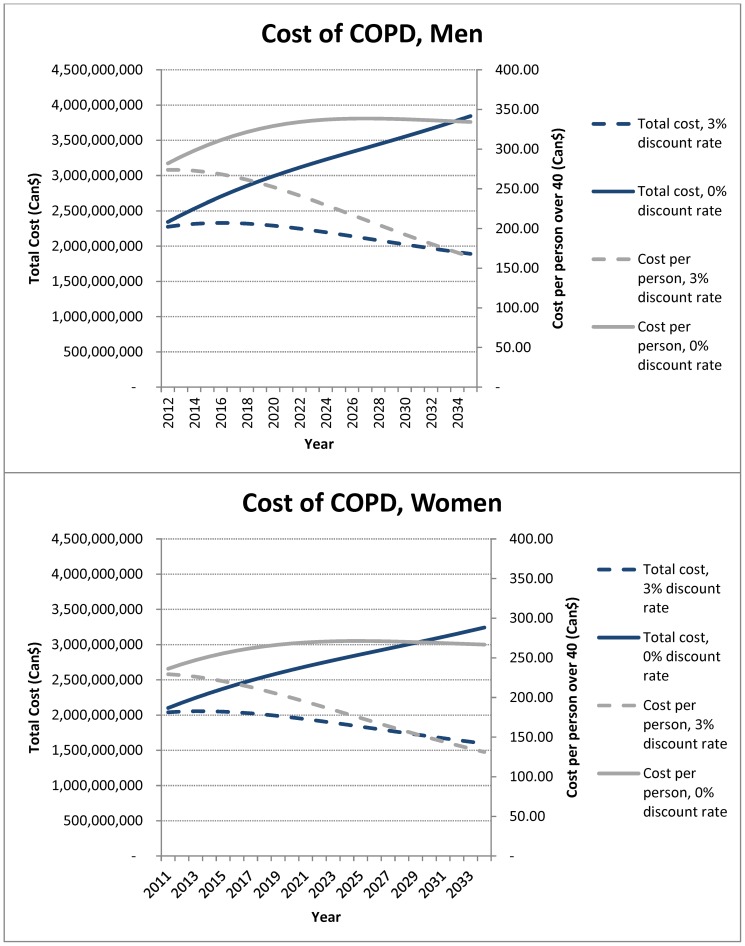
The projected changes in the costs related to COPD in Canada in men (A) and women (B) at various discount rates.

**Figure 4:**
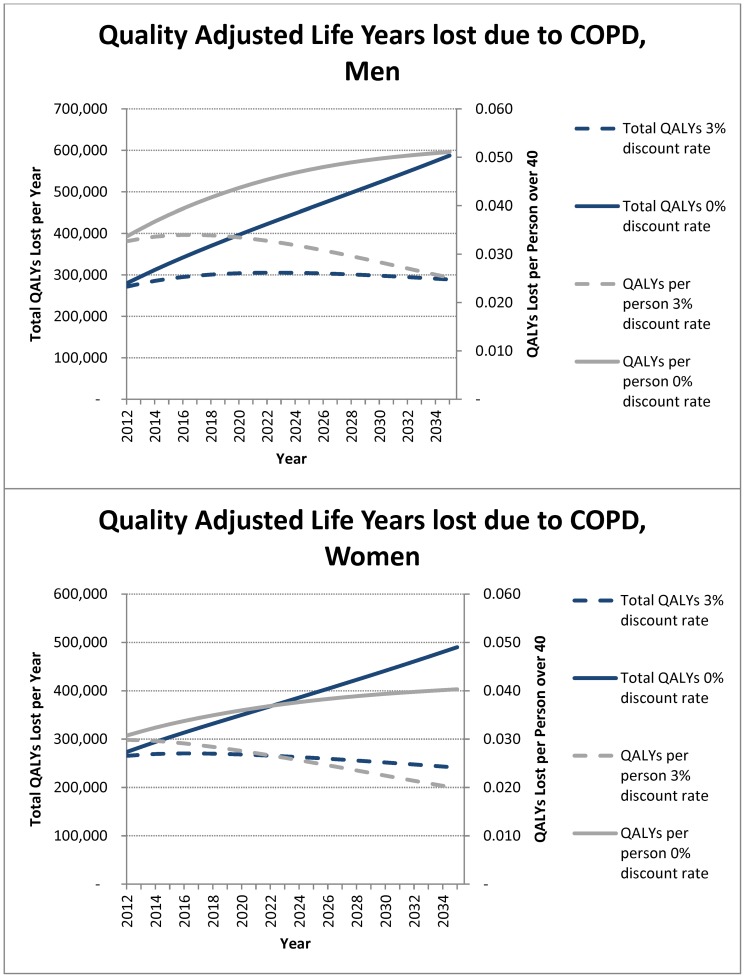
The projected changes in quality adjusted life years lost related to COPD in Canada in men (A) and women (B).

The population over the age of 40 will increase from about 17 million to about 24 million by 2035 in Canada. Importantly, during this time period, the population over the age of 70 will double to approximately 8 million (Appendix S1). The model also predicts that the number of COPD patients will increase from 3.45 million in 2011 to 5.83 million in 2035 (Figure 2). Based on these projections, the estimated annual societal cost of COPD in Canada is $4.52B in 2011, and will reach $3.61B ($7.33B undiscounted) per year in 2035 (Figure 3A and 3B). Over the next 25 years, COPD will be responsible for approximately $101.4B in societal costs ($147.5B undiscounted) and 12.9 million QALYs lost (19.0 million undiscounted) (Figures 4 A and B).

We also considered the proportion of COPD cases in the total population over 40 years as an indicator of change in the burden of COPD over the next 25 years in Canada. Our simulation showed that the prevalence of mild COPD will increase from 12.7% to 18.5% in men and from 10.0% to 14.1% in women over the next 25 years, while the prevalence of moderate COPD will decrease from 9% to 6.8% in men and from 5.7% to 5.0% in women and that for severe COPD will increase from 1% to 2.2% in men and remain unchanged at 1.9% in women over the course of analysis (Appendix S1).

**Figure 5:**
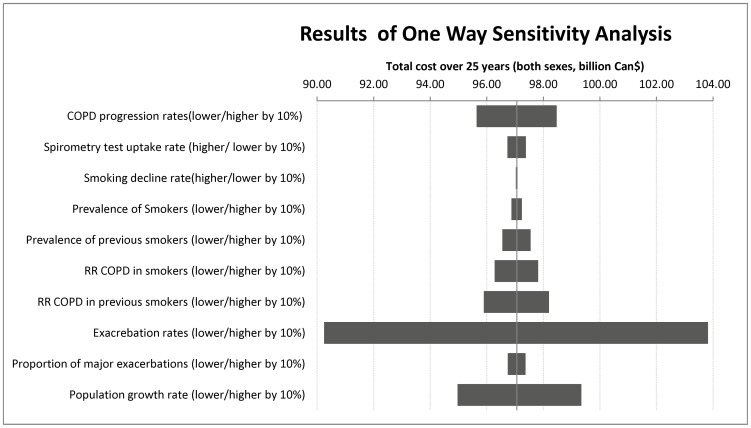
The results of one-way sensitivity analyses on the total cost related to COPD in Canada over the next 25 years.

The results of one way sensitivity analyses indicate that the total cost of COPD over next 25 years is most sensitive to the changes in the rates of exacerbation, followed by growth in the population, the relative risk of COPD in previous smokers, and rates of COPD progression in this order. Changes in the rate of spirometry testing and the relative risk of COPD among smokers had measurable but only a small effect on the total costs (Figure 5).

### 2. Cost-effectiveness of interventions

The interventions in this study are in discovery phase and therefore, no precise information about the intervention mechanisms or their costs are currently available. In this analysis, we set the cost for all three interventions at zero to facilitate cross-comparisons. As such, the estimated costs and QALYs represent the overall potential impact of these interventions on the burden of COPD without considering the cost of the interventions. The impact of all three interventions on total societal costs and QALYs is shown in Table 2. The total societal costs, which were Can$101.41 B in 2035 in the base case scenario, decreased to $101.20B, $100.23B, and $94.27 B with interventions I, II, and III, respectively, with each imparting 10% effect sizes. In the base case scenario, the total QALYs lost due to COPD were 12.93 million in 2035, which was reduced to 12.90, 12.60, 12.21 million with interventions I, II, and III, respectively, with each having 10% effect size (Table 2). Despite a large reduction in the number of smokers in Intervention I, the overall number of COPD cases declined only modestly (Table 2). We observed a large decline in the number of smokers with COPD as a result of intervention I. However, the number of previous smokers or never smokers with COPD increased over time.

**Figure 6:**
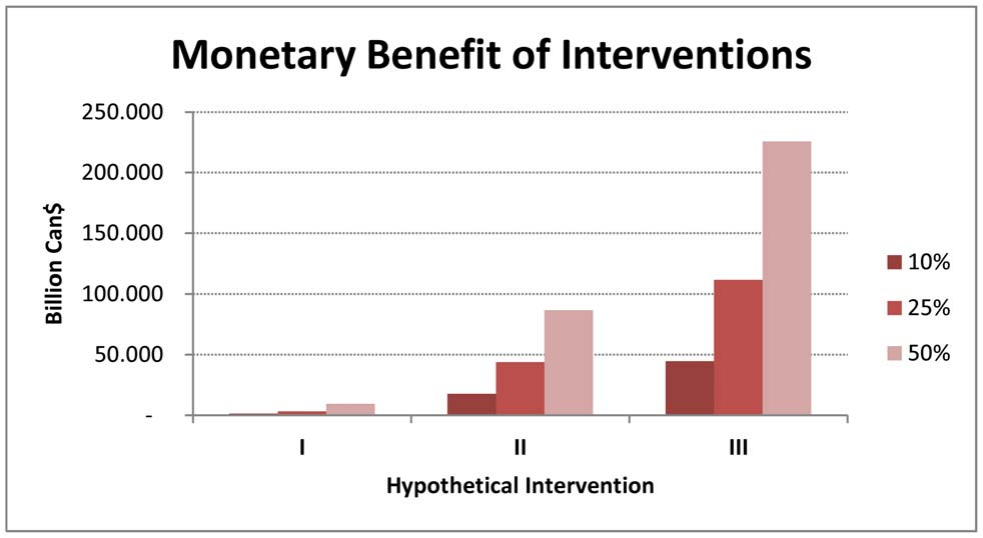
The estimated monetary benefit of each hypothetical intervention assuming different scenarios for effectiveness of these interventions in reducing smoking rates (intervention I), progression of disease (intervention II), and exacerbation rates (intervention II).

We calculated the monetary benefit of each intervention by converting QALYs into equivalent monetary benefit using a willingness to pay 50,000$ per QALY gained threshold and then adding cost savings associated with intervention. Comparison of estimated monetary benefits shows that intervention III has a very high potential for achieving benefits (Figure 6).

Because asthma is a risk factor for COPD and because the rates of asthma in children and adults are increasing in the Western World, we determined the impact of asthma on the costs and QALYs. However, over the next 25 years, the increased rates of asthma will have minimal impact on the costs or QALYs related to COPD, largely because of the significant lag time between asthma onset (which can be in childhood) to COPD diagnosis, which occurs in 50's and beyond and the uncertainty in the rate of progression of COPD in “asthmatics” over time (see Appendix S3)

## Discussion

Our findings confirm the enormous financial burden that COPD will pose over the next 25 years. The best strategy to reduce the growing financial and patient burden of COPD is to reduce exacerbations. Smoking cessation, while it is the cornerstone of COPD prevention, has only a modest effect in reducing COPD costs over the next 25 years in Western countries such as Canada. This is due to three important factors in COPD pathogenesis. First, there is considerable lag time between exposure and the onset of COPD. Although smoking rates have fallen considerably over the past decade, many ex-smokers will develop clinically relevant COPD with aging. Second, independent of smoking, the rates of COPD will increase with the increasing average age of the population [9]. Third, there is growing evidence that COPD may continue to progress despite smoking cessation [25]. Similarly, interventions (even very effective ones) that reduce the rate of decline in FEV_1_ will have a modest effect in reducing the economic and human burden of COPD over the next 25 years. This is because clinically relevant outcomes such as symptoms, hospitalizations or mortality correlate poorly with FEV_1_. Indeed, there are many patients with poor FEV_1_ who remain active and free of exacerbations, while there are others with well preserved FEV_1_ who are incapacitated by their disease and experience frequent exacerbations [14].

In our model, the most effective strategy was to target exacerbations. Exacerbations are expensive to treat and are associated with considerable morbidity and mortality. The current interventions for COPD reduce exacerbations by 20 to 30%. However, with improved understanding of the disease, in the near future, we may have therapies and diagnostic tests (to facilitate more “personalized care”) that may reduce exacerbations even further [26], [27]. If so, these interventions may have a large impact on the financial and human burden of COPD in the Western World.

To our best knowledge, this is the first dynamic population model for COPD in Canada. This model has compared the impact of population-based interventions that target different approaches to COPD management including primary (avoiding the development of disease), secondary (early disease detection), and tertiary (reducing the negative impact of an already established disease) prevention. The only other dynamic population model was developed by Hoogendoorn et al [28]–[30], which estimated the financial burden of COPD in the Dutch population between 2000 and 2025. They projected the incidence, prevalence, progression and costs of COPD in that period. To illustrate the applications of this model for effecting public health policy, Hoogendoorn et al compared the incremental cost and incremental QALYs of adding bupropion to counseling by general practitioners for smoking cessation. In a recent study using the same model [31], they also compared the cost effectiveness of three interventions (one pharmacologic, one on smoking cessation, and one on pulmonary rehabilitation interventions) on the costs of COPD over a 10 year time horizon.

While the current analysis relied on Canadian data and parameters, this selection was done for illustrative purposes and also because of the availability of robust COPD-related cost and epidemiological data. We believe that the overall conclusions are widely generalizable and would expect similar results in other jurisdictions with similar population demographics such as the US. In the US approximately 120,000 people die annually from COPD [20] and the total economic cost of COPD was estimated at $42.6 billion (USD) annually with more than 60% of these costs attributed to hospitalizations, which are largely driven by exacerbations [21].

There were some limitations to our study. There may be unpredicted changes in the future in various parameters that were modeled in this study, which may materially affect our projections. Age was divided into only four categories. Having smaller age groups could potentially increase the accuracy of the model. However, this approach is limited by the availability of data. For example, to generate accurate and robust cost data, accurate information on COPD, smoking and mortality rates for each age group is needed (Appendix S2). However, such data may not be available for small age ranges. Furthermore, we did not separate out GOLD class III from class IV disease because of the relatively small proportion of GOLD class IV patients in Canada (comprising ∼0.2% of adults 40 years of age or older; and ∼15% of combined class III/IV group), which made it difficult to estimate accurate cost data for this group of patients. Additionally, for parsimony, we assumed in our model that all UD and FN COPD cases had mild disease. We did this because there is a paucity of data on the costs generated and QALYs lost over time for this group of patients. While this is an over-simplification of the real world setting in which undiagnosed COPD patients can be found in all disease severity classes, our approach yielded a conservative estimate of the total costs of COPD and likely underestimated the true financial burden of this disease in Canada. Importantly, however, this approach does not materially affect the relative impact of the three hypothetical interventions on future costs of COPD in Canada. The use of probabilistic sensitivity analysis for dynamic models, unlike static models, entails additional complexities. For example, correlation between value of variables between different time points may lead to bifurcation [32], [33]. As such, similar to previous studies based on dynamic models [29], [30], we conducted extensive univariate sensitivity analyses to test the validity of the model and robustness of the results. However, we acknowledge that further work is warranted in this area. Finally, some studies suggested that incidence of asthma in early life has increased in recent years. The epidemiological link between asthma in early life years and COPD later in life has been evaluated by several studies and this relationship is indeed causal COPD rates will increase in future years [34], [35]. We conducted a sensitivity analysis to estimate the effect of a future increase in COPD due to asthma. Our analysis suggests that asthma-related increases in COPD costs will be relatively small over the next 25 years. A larger financial impact of asthma-related COPD will occur several decades later, owing to the lag time between childhood and even adult-onset asthma (occurring in 20's and 30's) and the onset of COPD, which generally occurs beyond age 50.

In conclusion, our results suggest that any intervention that can successfully reduce the number of exacerbations has an immediate and substantial impact on morbidity and costs of COPD and should be considered in conjunction with the ongoing efforts to reduce smoking rates.

## Supporting Information

Appendix S1 Additional model outputs.
(PDF)

Appendix S2 Description of model inputs and assumptions.
(PDF)

Appendix S3 The projected total costs and Quality-Adjusted Life Years lost related to COPD discounted at 3% per year in Canadian men to 2035 according to the effect of asthma on the overall COPD rates.
(PDF)
