# Supplementary material for: Future Impact of Various Interventions on the Burden of COPD in Canada: A Dynamic Population Model
Source: PLoS One. 2012 Oct 11;7(10):e46746. doi: 10.1371/journal.pone.0046746 (PMC3469627; doi:10.1371/journal.pone.0046746)
Supplement: Appendix S1 — Additional model outputs. (PDF) [file pone.0046746.s001.pdf]

Appendix S1. Additional model outputs

Projection of Canadian population 40 years of age or older over the next 25 years

The projected number of population based on smoking status over the next 25 years

The estimated changes in the prevalence of COPD

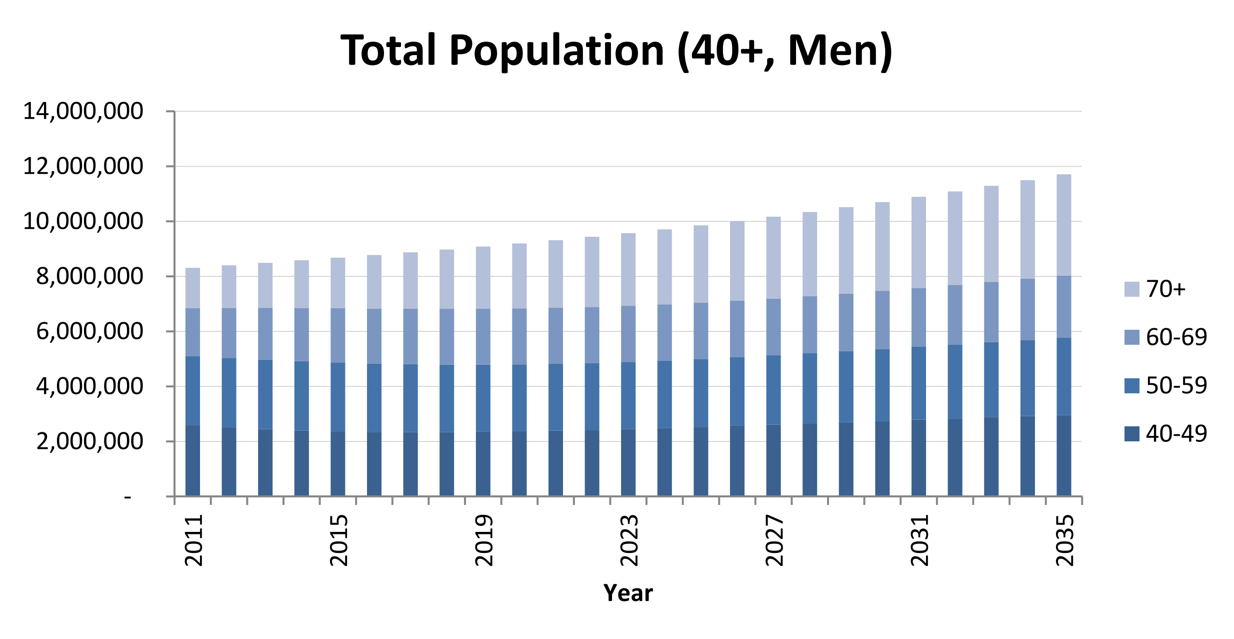
Total Population (40+, Men)
14,000,000
12,000,000
10,000,000
8,000,000
6,000,000
4,000,000
2,000,000
-
2011
2015
2019
2023
2027
2031
2035
Year
70+
60-69
50-59
40-49

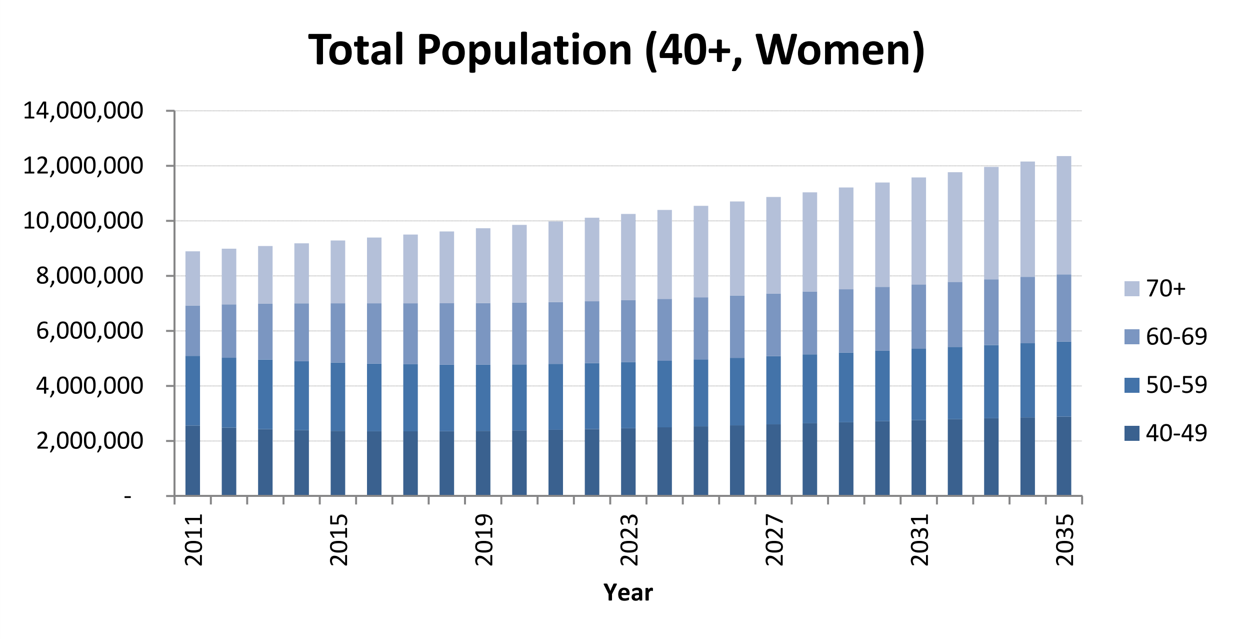
Total Population (40+, Women)
14,000,000
12,000,000
10,000,000
8,000,000
6,000,000
4,000,000
2,000,000
-
2011
2015
2019
2023
2027
2031
2035
Year
70+
60-69
50-59
40-49

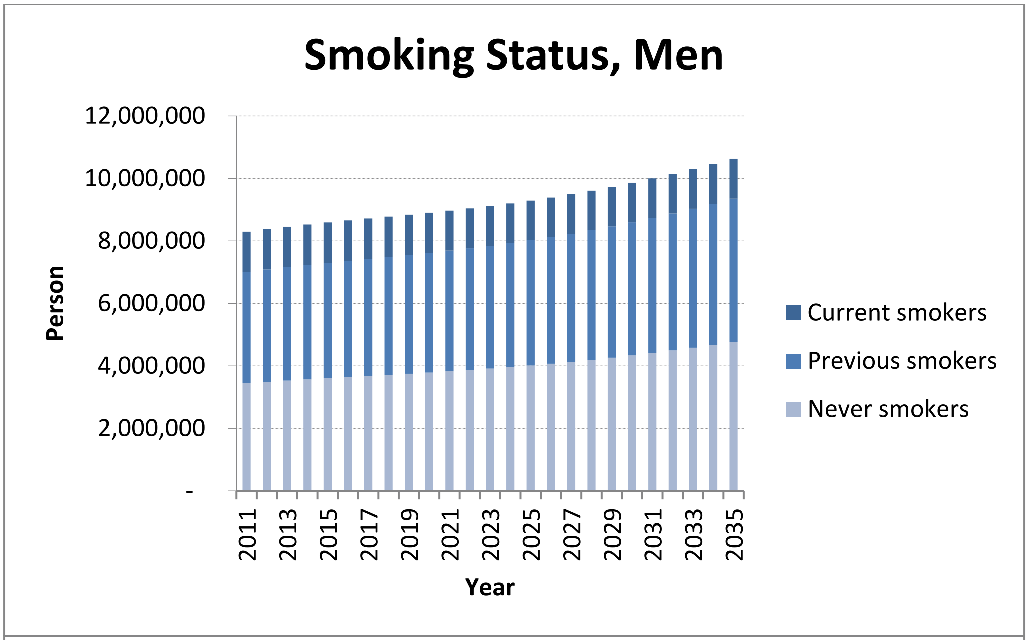
Smoking Status, Men
12,000,000
10,000,000
8,000,000
6,000,000
4,000,000
2,000,000
-
Person
2011
2013
2015
2017
2019
2021
2023
2025
2027
2029
2031
2033
2035
Year
Current smokers
Previous smokers
Never smokers

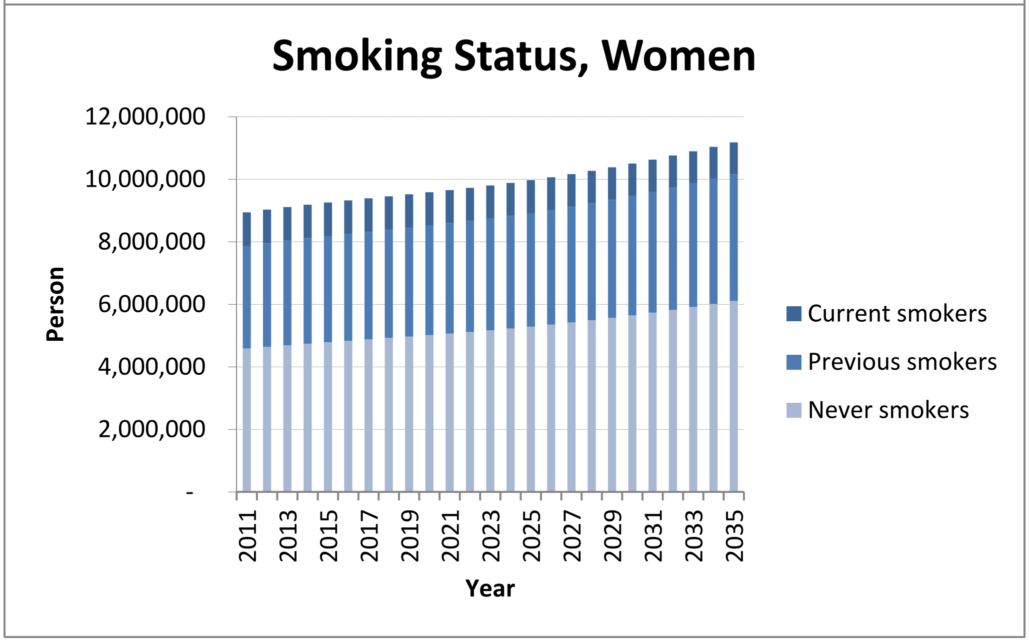
Smoking Status, Women
12,000,000
10,000,000
8,000,000
6,000,000
4,000,000
2,000,000
-
Person
2011
2013
2015
2017
2019
2021
2023
2025
2027
2029
2031
2033
2035
Year
Current smokers
Previous smokers
Never smokers

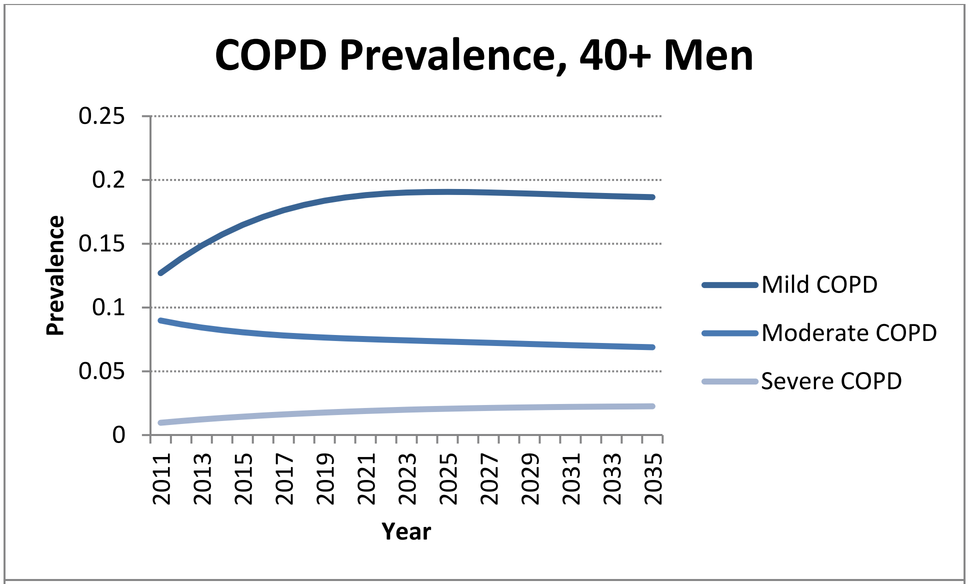
COPD Prevalence, 40+ Men
0.25
0.2
0.15
0.1
0.05
0
Prevalence
2011
2013
2015
2017
2019
2021
2023
2025
2027
2029
2031
2033
2035
Year
Mild COPD
Moderate COPD
Severe COPD

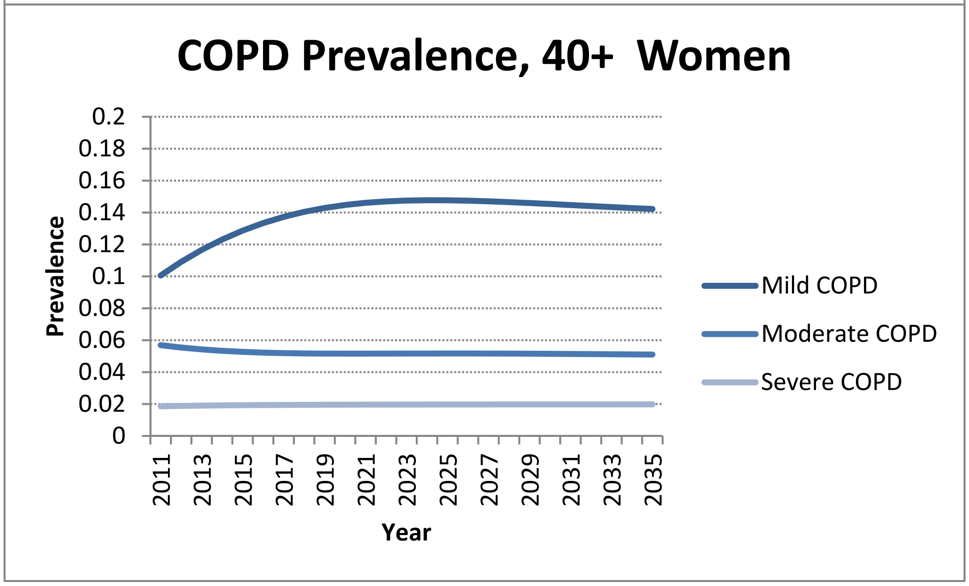
COPD Prevalence, 40+ Women
0.2
0.18
0.16
0.14
0.12
0.1
0.08
0.06
0.04
0.02
0
Prevalence
2011
2013
2015
2017
2019
2021
2023
2025
2027
2029
2031
2033
2035
Year
Mild COPD
Moderate COPD
Severe COPD
