# Supplementary material for: Future Impact of Various Interventions on the Burden of COPD in Canada: A Dynamic Population Model
Source: PLoS One. 2012 Oct 11;7(10):e46746. doi: 10.1371/journal.pone.0046746 (PMC3469627; doi:10.1371/journal.pone.0046746)
Supplement: Appendix S2 — Description of model inputs and assumptions. (PDF) [file pone.0046746.s002.pdf]

Appendix S2. Description of model inputs and assumptions

Interpolation of rates in subgroups

The subgroups in the dynamic model were defined based on smoking status (current smokers, previous smokers, never smokers), COPD stage (no COPD, stage i, stage ii, stage iii or higher), age group (40-49, 50-59, 60-69, ≥70 years), and sex (women, men). Therefore, input parameters in the model (e.g. prevalence rates, mortality rates, exacerbations) had to be defined for each subgroup (3*4*4*2=96 possible subgroups). For example, prevalence rates for different stages of COPD and for different age groups are available for men and women (Table 4, Buist et al.[1]). In addition, prevalence rates for different stages of COPD based on smoking status are also available for men and women (Table 3, Buist et al [1]). However, prevalence rates stratified by smoking status AND age group are not directly available and had to be estimated. Therefore, we used a simple approximation method to interpolate the rates that were not directly available in the literature. This interpolation method was based on relative risk of COPD based on smoking status and also ensured that the weighted average of COPD rates within each age stratum adds up to the observed marginal rate in that stratum.

Similar method was used to interpolate mortality rates in table 1. According to the projections of Statistics Canada, total number of all-cause deaths will increase from 243,500 in 2010 to 375,400 in 2036 [2]. We used Canadian life tables to estimate age specific mortality rates for each age category (40-49, 50-59, 60-69, ≥70 years) using piecewise linear approximations. Then, we calculated the approximate number of total deaths that are attributable to each age category. The relative risk of

background mortality has been reported as 1.7, 1.2, and 1 for current smokers, ex-smokers, and never smokers, respectively[3]. These rates, in conjunction with prevalence of smokers (13.25%), previous smokers (39.4%), and never smokers (47.3%) were used to interpolate the number of deaths given smoking status within each age group (using the same approach that was explained above). Mortality rates are expected to decrease for population over 40 years old, as a result of prolonged life expectancy. Therefore, the model accounted for the declining mortality rates in future years.

Calculation of direct and indirect costs

The estimated costs by Spencer et al[4] were used to calculate direct cost associated with a COPD patient at different stages of COPD. We adjusted those costs (cost of maintenance, minor exacerbations, and major exacerbations) using Consumer Price Index (CPI) such that they can represent 2011 Canadian currency value. Overall, direct cost for a patient with mild, moderate, or severe COPD consisted of maintenance costs and costs due to minor/major exacerbations. We assumed that maintenance costs are pertinent to all patients in a given severity stage. However, exacerbations occur only in fraction of those patients and as such, we used frequency of minor/major exacerbations per each severity stage to calculate expected cost of exacerbations for a patients in the given severity stage. The indirect cost of COPD accounts for approximately 20%, 33%, and 45% of total costs for mild, moderate, and severe COPD patients respectively[5]. Hence we assumed that indirect costs constitute 25%, 50%, and 83% of direct costs for mild, moderate, and severe COPD, respectively.

Progression rates between different stages of COPD

Annual decline rates in lung function were adapted from the estimated rates in Hoogendoorn et al [6]. Based on those estimates, annual decline in FEV% was 0.83 and 1.20 for mild and moderate COPD respectively in non smoker males, and was 1.16 and 1.54 respectively for smoker males. Those numbers were 0.79 and 1.17 for mild and moderate COPD in nonsmoker females and 1.13 and 1.51 in mild and moderate smoker females.

We used the lung age curves- that describe the relationship between the changes in the lung function and age by smoking status[7] - to estimate the average progression times (Table 1) for moving from mild to moderate and from moderate to severe COPD given the abovementioned decline rates in FEV.

1 Buist, A. S. *et al.* International variation in the prevalence of COPD (the BOLD Study): a population-based prevalence study. *Lancet* **370**, 741-750, doi:S0140-6736(07)61377-4 [pii]
10.1016/S0140-6736(07)61377-4 [doi] (2007).

2 Population Projections for Canada, Provinces and Territories 2009 to 2036. *Statistics Canada* (2010).

3 Rosenbaum, W. L., Sterling, T. D. & Weinkam, J. J. Use of multiple surveys to estimate mortality among never, current, and former smokers: changes over a 20-year interval. *Am J Public Health* **88**, 1664-1668 (1998).

4 Spencer, M., Briggs, A. H., Grossman, R. F. & Rance, L. Development of an economic model to assess the cost effectiveness of treatment interventions for chronic obstructive pulmonary disease. *Pharmacoeconomics* **23**, 619-637, doi:2368 [pii] (2005).

5 Chapman, K. R., Bourbeau, J. & Rance, L. The burden of COPD in Canada: results from the Confronting COPD survey. *Respir Med* **97**, S23-31 (2003).

6 Hoogendoorn, M., Rutten-van Molken, M. P., Hoogenveen, R. T., Al, M. J. & Feenstra, T. L. Developing and applying a stochastic dynamic population model for chronic obstructive pulmonary disease. *Value Health* **14**, 1039-1047, doi:10.1016/j.jval.2011.06.008 (2011).

7 Parkes, G., Greenhalgh, T., Griffin, M. & Dent, R. Effect on smoking quit rate of telling patients their lung age: the Step2quit randomised controlled trial. *BMJ* **336**, 598-600, doi:bmj.39503.582396.25 [pii]
10.1136/bmj.39503.582396.25 [doi] (2008).
