# Supplementary material for: Future Impact of Various Interventions on the Burden of COPD in Canada: A Dynamic Population Model
Source: PLoS One. 2012 Oct 11;7(10):e46746. doi: 10.1371/journal.pone.0046746 (PMC3469627; doi:10.1371/journal.pone.0046746)

Appendix S3: The projected total costs and Quality-Adjusted Life Years lost related to COPD discounted at 3% per year in Canadian men to 2035 according to the effect of asthma on the overall COPD rates

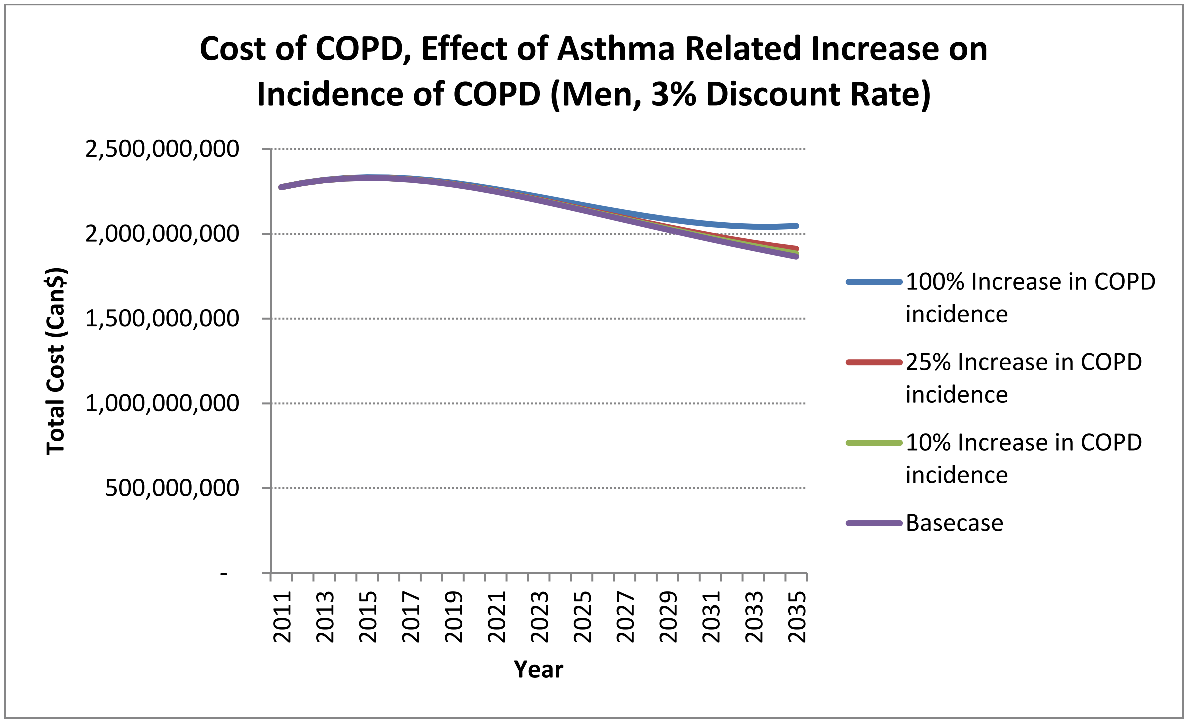

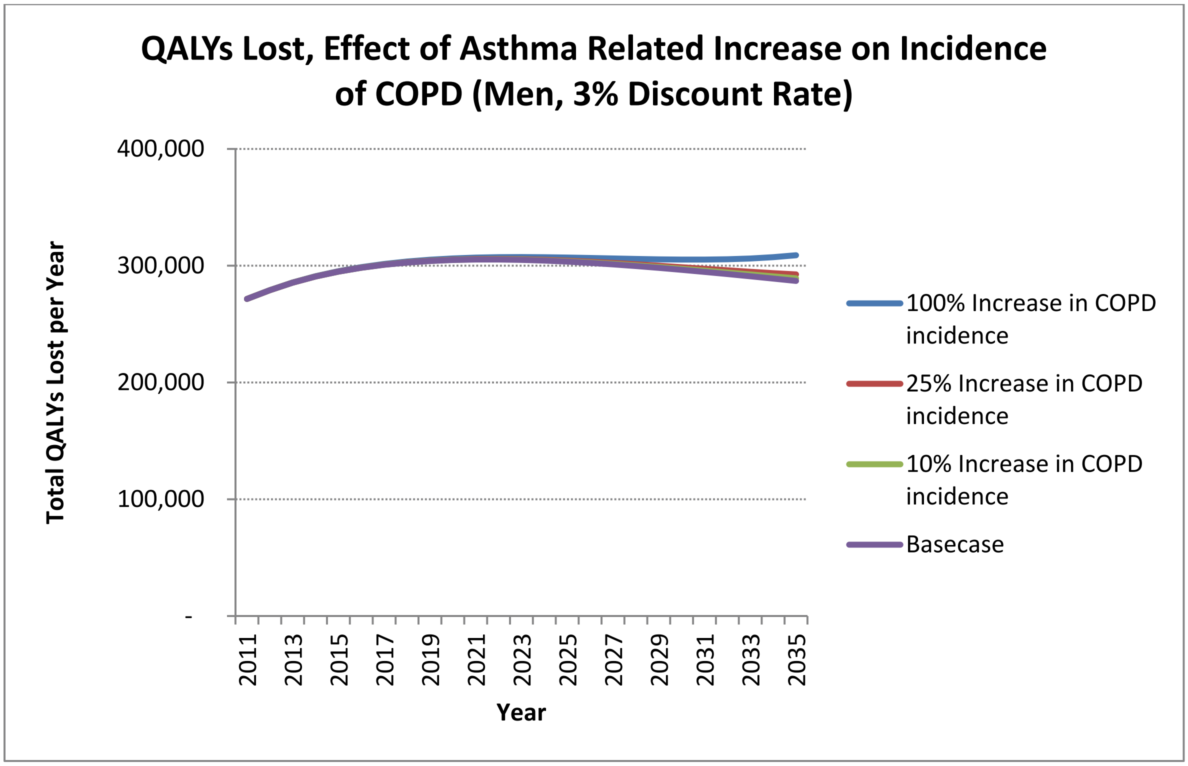

Supplement: Appendix S3 — The projected total costs and Quality-Adjusted Life Years lost related to COPD discounted at 3% per year in Canadian men to 2035 according to the effect of asthma on the overall COPD rates. (PDF) [file pone.0046746.s003.pdf]
